# A plastic corticostriatal circuit model of adaptation in perceptual decision making

**DOI:** 10.3389/fncom.2013.00178

**Published:** 2013-12-10

**Authors:** Pao-Yueh Hsiao, Chung-Chuan Lo

**Affiliations:** ^1^Institute of Systems Neuroscience, National Tsing Hua UniversityHsinchu, Taiwan; ^2^Department of Life Science, National Tsing Hua UniversityHsinchu, Taiwan

**Keywords:** perceptual decision, adaptation, speed-accuracy tradeoff, neural network model, corticostriatal circuit, spike-timing dependent plasticity

## Abstract

The ability to optimize decisions and adapt them to changing environments is a crucial brain function that increase survivability. Although much has been learned about the neuronal activity in various brain regions that are associated with decision making, and about how the nervous systems may learn to achieve optimization, the underlying neuronal mechanisms of how the nervous systems optimize decision strategies with preference given to speed or accuracy, and how the systems adapt to changes in the environment, remain unclear. Based on extensive empirical observations, we addressed the question by extending a previously described cortico-basal ganglia circuit model of perceptual decisions with the inclusion of a dynamic dopamine (DA) system that modulates spike-timing dependent plasticity (STDP). We found that, once an optimal model setting that maximized the reward rate was selected, the same setting automatically optimized decisions across different task environments through dynamic balancing between the facilitating and depressing components of the DA dynamics. Interestingly, other model parameters were also optimal if we considered the reward rate that was weighted by the subject's preferences for speed or accuracy. Specifically, the circuit model favored speed if we increased the phasic DA response to the reward prediction error, whereas the model favored accuracy if we reduced the tonic DA activity or the phasic DA responses to the estimated reward probability. The proposed model provides insight into the roles of different components of DA responses in decision adaptation and optimization in a changing environment.

## Introduction

To ensure survivability in complex environments, animals need to adapt to environments that may change with time. For example, when making decisions, animals have to choose a proper strategy that is optimal for the current situation, such as making quick but inaccurate decisions vs. making slow but accurate decisions. However, whether a decision strategy is proper or not may be subject- and environment-dependent. Indeed, at the behavioral level, speed-accuracy tradeoff (SAT) has been demonstrated in humans and animals in various decision experiments (Schouten and Bekker, 1967; Wickelgren, 1977; Palmer et al., 2005; Chittka et al., 2009; Balci et al., 2010). Although it has been suggested that a strategy can be optimized by maximizing the reward rate (Gold and Shadlen, 2002; Lo and Wang, 2006), several studies have demonstrated cases in which subjects adopt strategies that are faster (but less accurate) or slower (but more accurate) than the ideal one that maximizes the reward rate (Maddox and Bohil, 1998; Chittka et al., 2009; Bogacz et al., 2010).

At the neuronal level, numerous studies have shown correlations between the complex responses of dopamine (DA) neurons and reward-related information in decision making (Schultz, 2000, 2002; Arias-Carrión et al., 2010; de Lafuente and Romo, 2011). Furthermore, the responses of DA neurons do not simply depend on the presence of rewards, but also on various task-related factors such as the expected probability and magnitude of rewards and reward prediction errors (Hollerman and Schultz, 1998; Schultz, 1999; Kawagoe et al., 2004; Takikawa et al., 2004; Nomoto et al., 2010). However, how the DA system contributes to the mechanisms underlying the differential strategy chosen, and how the animals adapt their decision strategies to changes in the environment, remain unclear.

At the theoretical level, a number of mathematical models and neural network models have been proposed to account for decision behavior (Ratcliff, 1978; Bogacz et al., 2006; Lo and Wang, 2006; O'Reilly and Frank, 2006; Simen et al., 2006; Bogacz and Gurney, 2007; Roxin and Ledberg, 2008; Wang, 2008; Cohen and Frank, 2009; Deco et al., 2009; Hong and Hikosaka, 2011). In particular, a spiking neural network model consisting of an attractor cortical network for information accumulation (Wang, 2002, 2008; Wong et al., 2007; Wang et al., 2013) and a cortico-basal ganglia circuit mediating the decision threshold (Lo and Wang, 2006) have suggested that the threshold can be modulated by the strength of the corticostriatal pathway. Hence, this has provided insights into how SAT may be implemented in the neural circuits that perform perceptual decisions. This prediction has been subsequently supported by human functional magnetic resonance imaging (fMRI) experiments (Forstmann et al., 2010).

Despite this progress, we still lack an integrated model, with sufficient biological detail, that illustrates the neuronal mechanisms underlying how individual subjects choose their decision strategies differently, how their decision strategies adapt to changing environments, and how detailed DA neuron activity, including responses to expected reward probability or reward prediction errors, may play a role in behavioral flexibility. Although reward-dependent plasticity has been incorporated in a number of rate-based neural network models to account for various flexible behaviors (Simen et al., 2006; Cohen and Frank, 2009; Rao, 2010; Bogacz and Larsen, 2011; Wiecki and Frank, 2013), most of the models either do not address how the subjects adapt their decisions to changing environments, or were built based on relatively abstract learning rules that do not include complex real-time dopamine dynamics and spike-time based plasticity that has been observed empirically (Suaud-Chagny et al., 1995; Bi and Poo, 1998; Shen et al., 2008; Nomoto et al., 2010). Some of the models have been designed for simulating action-selection and executive control, which may involve very different mechanisms from the perceptual discrimination that we studied here.

We present a spiking neural circuit model that integrates a spiking cortico-basal ganglia model (Lo and Wang, 2006) and a DA system with complex dynamics that is consistent with empirical observations (Suaud-Chagny et al., 1995; Bi and Poo, 1998; Shen et al., 2008; Nomoto et al., 2010). We demonstrate that different decision strategies that are adopted by different animals can all be optimal in their own subjective sense which is weighted by their individual preferences for speed or accuracy. Furthermore, the interplay between the different temporal components of the DA dynamics supports the adaptation of the optimal strategies in response to changes in the environment.

## Materials and methods

### The basic hypothesis and logic of the model

In the present study, we did not investigate how a neural network can learn to achieve an optimal decision strategy, which has been addressed in various modeling studies, and we accepted that an optimal decision can be achieved by learning. Instead, we hypothesized that there is not just one, but many different optimal decision strategies that are weighted by individual's preferences for speed or accuracy. Our goal was to study how preferences were influenced by the detailed activity of the DA system and how dynamic balancing between different DA effects helped the subjects to adapt to changes in the environment when making perceptual decisions.

### The behavioral task: random-dot motion discrimination

In order to investigate adaptive behavior in perceptual decision making and its optimization, we used the reaction-time version of the random-dot motion discrimination task (Newsome et al., 1989; Shadlen and Newsome, 1996; Roitman and Shadlen, 2002). In the task, a subject watches a display of randomly moving dots with a center fixation point and two saccade targets that are located on the two sides of the screen. A small fraction of the dots move coherently in one of two possible directions (right or left in our simulations), and the subject is required to determine the direction of the coherent movement while fixating on the center of the screen. The subject needs to make a saccade to the corresponding target (right target for right coherent movements and left target for the left coherent movements) as soon as a decision is reached.

The percentage of dots that move coherently is defined as the coherence level (*c*′) or motion strength, and it represents the amount of evidence that is available to the subject during the decision process. Motion discrimination is easier in trials with a strong, compared to a weak, motion strength. We tested our model with two conditions: easy and difficult. The easy condition consisted only of trials with *c*′ levels of 12.8, 25.6, and 51.2%, while the difficult condition consisted only of trials with *c*′ levels of 3.2, 6.4, and 12.8%. The trials of the different coherence levels in each condition were pseudorandomly distributed with equal probability. The reaction time was defined as the interval between the start of the sensory input and the time when the subject made a saccade.

### Single neuron model

Neurons in the circuit model were simulated with the conductance-based leaky integrate-and-fire model that is described as follows.

The membrane potential *V(t)* obeys the following equation:

$$C_{m}\frac{dV(t)}{dt}=-g_{L}\left(V(t)-V_{L}\right)-I_{\text{syn}}(t),$$

where *C*_*m*_ is the membrane capacitance, *g*_*L*_ is the leak conductance, *V*_*L*_ is the resting potential, and *I*_syn_ is the total synaptic current. When the membrane potential *V*(*t*) of each neuron reaches the threshold *V*_threshold_ = −50 mV, a spike is emitted, and *V(t)* is set to the reset potential *V*_reset_ = −55 mV for a refractory period *T*_*r*_ = 2 ms. For inhibitory neurons, we used the following parameters: *C*_*m*_ = 0.2 nF, *g*_*L*_ = 20 nS, and *V*_*L*_ = −70 mV. For excitatory neurons, we used *C*_*m*_ = 0.5 nF, *g*_*L*_ = 25 nS, and *V*_*L*_ = −70 mV. *I*_syn_ is the total synaptic current and is given by:

$$\begin{array}{l} I_{\text{syn}}(t)=g_{\text{AMPA}}s_{\text{AMPA}}(t)\left(V(t)-V_{E}\right)\\ \;+\frac{g_{\text{NMDA}}s_{\text{NMDA}}(t)\left(V(t)-V_{E}\right)}{1+[Mg^{2+}]e^{-0.062V(t)/3.57}}\\ \;+g_{\text{GABA}}s_{\text{GABA}}(t)\left(V(t)-V_{I}\right) \end{array}$$

where (*V*_*E*_ (=0 mV) and *V*_*I*_ (= −70 mV) are the reversal potentials, [Mg^2+^] (= 1.0 mM) is the extracellular magnesium concentration, *g* is the synaptic efficacy, and *s* is the gating variable. The subscripts in g and s denote the receptor type. The gating variables are described by

$$\frac{ds(t)}{dt}=\sum_{k}\delta \left(t-t^{k}\right)-\frac{s}{\tau }$$

for AMPA and GABA receptors and

$$\frac{ds(t)}{dt}=\alpha \left(1-s(t)\right)\sum_{k}\delta \left(t-t^{k}\right)-\frac{s}{\tau }$$

for NMDA receptors, where τ = 2 ms for AMPA, 100 ms for NMDA, and 5 ms for GABA. δ(*t* − *t*^*k*^) is the delta function, and *t*^*k*^ is the time of the *k-*th pre-synaptic spike. The differential equations were solved numerically by the first-order Euler method with a time step of 0.1 ms.

### The cortico-basal ganglia neural circuit of decision making

**Figure 1:**
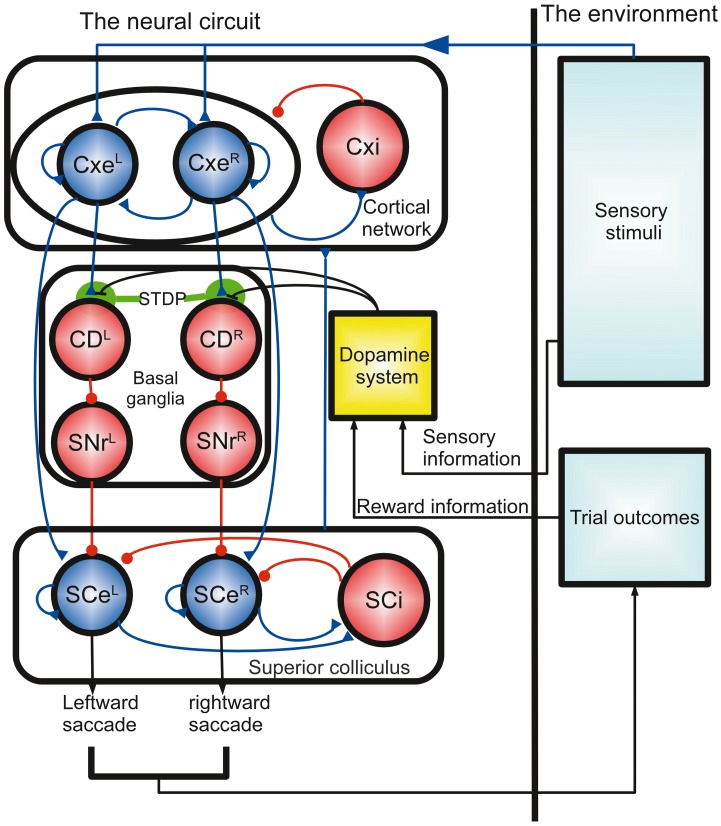
**Schematics of the computational model of decision optimization and adaptation for the random-dot direction discrimination task**. The model is described by a close loop process that involves a neural circuit based on a dopamine system and a previously described cortico-basal ganglia circuit model (Lo and Wang, 2006). The neural circuit receives sensory input, accumulates sensory evidence (random dot movements), detects the threshold crossing, and then makes a decision (saccadic eye movements). The outcome of the decision determines the reward and thereby influences the dopamine system. The dopamine activity modulates the decision process by changing the Cx-CD synaptic strength through spike-timing dependent plasticity (STDP). Each circle represents a population of leaky integrate-and-fire neurons, and the dopamine system is a functional unit that is modeled by several equations. Cx, cortex; Cxe, excitatory cortical pyramidal neurons; Cxi, inhibitory cortical interneurons; CD, caudate nucleus; SNr, substantia nigra pars reticulata; SCe, superior colliculus excitatory neurons; SCi, superior colliculus inhibitory neurons. The L and R superscripts denote the neural populations responding to the left and right stimuli, respectively.

The model of the corticobasal ganglia neural circuit used in the present study was developed based on a previously described model (Lo and Wang, 2006), which consists of a cortical (Cx) circuit, a superior colliculus (SC) circuit, and a basal ganglia (BG) circuit (Figure 1). All parameters in the previous model (Lo and Wang, 2006) were preserved except for two changes made to CD neuron-related parameters due to the inclusion of the synaptic plasticity in the present model. First, we added NMDA receptors to the Cx-to-CD synapses and reduced the conductance for the AMPA-mediated current on the same synapse accordingly. Second, we increased the background noise for CD neurons in order to produce a baseline firing rate of several spikes per second. The small baseline activity is important for maintaining plasticity throughout the course of a trial. See Table 1 for the parameters that were used in the circuit model. Three major neural processes described below are involved in the model:

1. Winner-take-all competition for sensory signals in the cortical network: After the stimulus onset, two signals representing the amount of leftward and rightward dot movements [presumably from the visual MT area (Britten et al., 1993)] project to the neural populations Cxe^L^ and Cxe^R^ in the Cx, respectively. The two decision populations, Cxe^L^ and Cxe^R^, compete with each other through the inhibitory interneurons in Cxi. As a consequence, the decision population receiving the stronger sensory input has a higher chance to accumulate its activity (population firing rate) and suppress the other (Wang, 2002). The activity of the two decision populations are sent to the downstream regions (SC and BG) for further computation. There is a non-selective background population, CxeBg, that mimics neurons that are selective for directions other than the two forced-choice alternatives or to other stimuli that are irrelevant to the present study.
2. All-or-nothing motor output in the SC: The neural circuit of the SC is similar to that of the Cx but with two important differences. First, the feedback excitation in SCe^L^ and SCe^R^ and the lateral inhibition between SCi and SCe^R^/SCe^L^ are much stronger than those in Cx. Second, the lateral inhibition is endowed with short-term synaptic facilitation, as described in Lo and Wang (2006). These differences allow neurons in the SCe^R^ or SCe^L^ to develop a strong burst of spikes lasting for about 50 ms in response to input from the Cx. The activity resembles what has been observed in burst neurons in the SC of monkeys during various saccadic eye movement experiments (Munoz and Wurtz, 1995; Sparks, 2002). In the model, the saccadic eye movement was triggered, or a decision was made, when the population firing rate of SCe^R^ or SCe^L^ reached the threshold of 60 spikes/s. In addition to the excitatory input from the Cx, SCe^R^, and SCe^L^ were constantly suppressed by feed-forward inhibition from the BG. Therefore, the Cx could only activate the SCe when it was disinhibited by the BG.
3. Threshold-crossing detection and disinhibition: The SNr^L^ and SNr^R^ in the BG constantly exhibit inhibition over the SCe^L^ and SCe^R^ in the SC, respectively. This inhibition can be removed by GABAergic input from upstream caudate nuclei (CD)^L^ and CD^R^, which in turn receive inputs from the decision neurons in the Cx. Therefore, when the activity in the Cxe^L^ (or Cxe^R^) is strong enough to activate CD^L^ (or CD^R^), the activated CD^L^ (or CD^R^) disinhibits the SC through the SNr^L^ (or SNr^R^) and allows SCe^L^ (or SCe^R^) to respond to input from the Cxe^L^ (or Cxe^R^), thus producing a saccadic eye movement. The activation of the CD^L^ or CD^R^ indicates the detection of the crossing of the decision threshold. Hence, by varying the corticostriatal (Cx-CD) synaptic strength, we could change the level of the decision threshold. Specifically, a stronger Cx-CD strength resulted in a lower decision threshold, while a weaker strength resulted in a higher decision threshold.

**Table 1:** **Parameters of the circuit model**.

**Population name**	**Number of neurons**	**Membrane capacitance (nF)**	**Background input to AMPA receptors (frequency in Hz/conductance in nS)**	**Target population (receptor type/strength in nS)**
SCe^L^	250	0.5	1280/0.19	SCe^L^ (N/1.5), SCi (N/0.7), Cxi (N/0.11), Cxe^L^ (N/0.05), Cxe^R^ (N/0.05)
SCe^R^	250	0.5	1280/0.19	SCe^R^ (N/1.5), SCi (N/0.7) Cxi (N/0.11), Cxe^L^ (N/0.05), Cxe^R^ (N/0.05),
SCi	250	0.2	1280/2.0	SCe^L^ (G/2.5), SCe^R^ (G/2.5)
SNr^L^	250	0.5	3440/2.0	SCe^L^ (G/2.5)
CD^L^	250	0.5	400/8.0	SNr^L^ (G/0.6)
SNr^R^	250	0.5	3440/2.0	SCe^R^ (G/2.5)
CD^R^	250	0.5	400/8.0	SNr^R^ (G/0.6)
CxeBG	1120	0.5	2400/2.1	CxeBG (A/0.05), CxeBG (N/0.165), Cxe^L^ (A/0.043825), Cxe^L^ (N/0.14462), Cxe^R^ (A/0.043825), Cxe^R^ (N/0.14462) Cxi (A/0.04), Cxi (N/0.13)
Cxe^L^	240	0.5	2400/2.1	SCe^L^ (A/3.5), CD^L^ (N/0.2), CD^L^ (A/0.12), CxeBG (A/0.05), CxeBG (N/0.165), Cxe^L^ (A/0.085), Cxe^L^ (N/0.2805), Cxe^R^ (A/0.043825), Cxe^R^ (N/0.14462), Cxi (A/0.04), Cxi (N/0.13)
Cxe^R^	240	0.5	2400/2.1	SCe^R^ (A_3.5), CD^R^ (N_0.2), CD^R^ (A_0.12), CxeBG (A_0.05), CxeBG (N_0.165), Cxe^L^ (A_0.043825), Cxe^L^ (N/0.14462), Cxe^R^ (A/0.085), Cxe^R^ (N/0.2805), Cxi (A/0.04), Cxi (N/0.13)
Cxi	400	0.2	2400/1.62	CxeBG (G/1.3), Cxe^L^ (G/1.3) Cxe^R^ (G/1.3), Cxi (G/1.0)

In the model, the mean spike rate μ of each input to Cxe^L^/Cxe^R^ depended on the motion strength of the stimulus linearly and followed the equations: μ = μ_0_ + μ_A_ × *c*′ for the direction of the coherent motion and μ = μ_0_ − μ_B_ × *c*′ for the opposite direction. The variable μ_0_ (40 Hz) was the baseline input for the purely random motion, *c*′ (between 0 and 100%) was the coherence level that characterized the stimulus motion strength, and μ_A_ (120 Hz) and μ_B_ (40 Hz) were the factors of proportionality, based on empirical observations (Britten et al., 1993).

In each trial, the reaction time was calculated by adding the decision time with a 250 ms non-decision time. The decision time was the time interval between the stimulus presentation to the model and the crossing of the response threshold in SCe neurons. The non-decision time represented the time that was needed for the sensory processing and the motor output that were not modeled in the present study.

In a previous study (Lo and Wang, 2006), we demonstrated that the major determining factor of the threshold is the Cx-CD synaptic strength and that there exists an optimal strength that results in the maximum reward rate (averaged number of rewards acquired per second). The optimal strength was different for the different task conditions. In the present study, the Cx-CD synapses were endowed with DA-dependent plasticity, and we investigated how the neural circuit adapted to the changing task conditions and remained optimized through the action of the DA system.

### Dopamine-dependent synaptic plasticity

**Figure 2:**
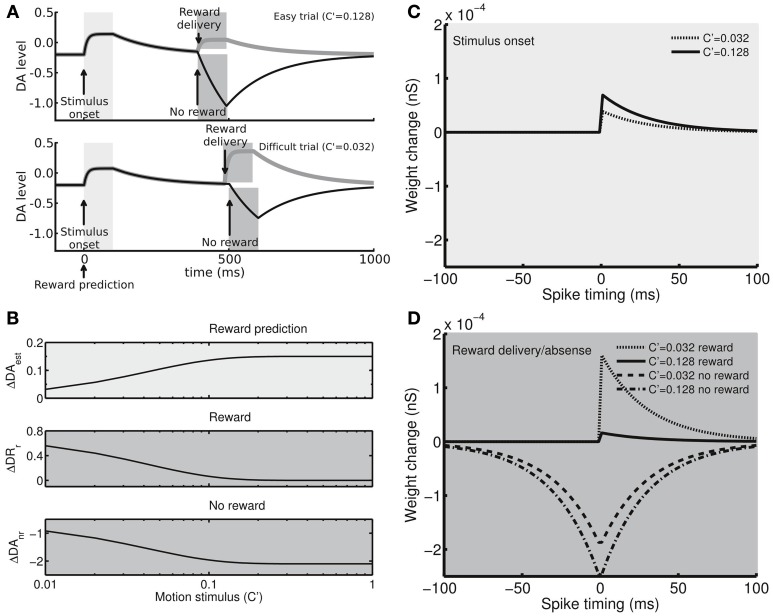
**The dopamine (DA) system and spike-timing dependent plasticity. (A)** Example time courses showing that the DA levels (with respect to the neutral level, in arbitrary units) exhibit phasic activity in response to the stimulus presentation (light gray regions) and reward delivery/absence (dark gray regions). In an easy trial (top), the DA levels strongly respond to the stimulus presentation due to the large estimated reward probability but only result in a moderate increase in response to the reward delivery due to the small reward prediction error. In an erroneous trial, the absence of reward strongly suppresses the DA levels for the large and negative reward prediction error. In contrast, in a difficult trial (bottom), the DA levels increase moderately in response to the stimulus onset due to the small estimated reward probability, while the levels exhibit strong (or weak) responses for reward delivery (or absence). **(B)** The peak levels of the DA phasic responses to the stimulus presentation (top), reward delivery (middle), and reward absence (bottom) as functions of the difficulty of the task (stimulus motion strength). The responses to the stimulus presentation correlate with the estimate of reward probability, whereas the responses to the reward delivery and absence correlate with the reward prediction error. **(C)** Example STDP kernels (synaptic weight change Δ*w* as a function of pre- and post-synaptic spike time difference) at the peak DA levels for the stimulus presentations. **(D)** Example STDP kernels at the peak/bottom DA levels for the reward delivery/absence.

Previous studies have demonstrated that the activity of DA neurons in the substantia nigra pars compacta (SNc) correlates with reward prediction error as well as with the stimuli that predict rewards (Hollerman and Schultz, 1998; Schultz, 1999; Kawagoe et al., 2004; Arias-Carrión et al., 2010; Nomoto et al., 2010). In the present study, we studied the behavior of fully trained subjects and assumed that they had developed a sense of the correlation between the performance (percentage correct) and the task difficulty (stimulus motion strength) Equation (1) below. Therefore, the subjects were able to estimate the probability of receiving a reward at the onset of the motion stimulus and thereby to evaluate the reward prediction error at the time of reward delivery/absence. Based on the hypothesis, the DA system in our model responded to the onset of the motion stimulus with a magnitude that correlated with the estimated reward probability as well as to the reward delivery/absence with a magnitude that correlated with the reward prediction error (Figure 2A). The hypothesized DA activity was consistent with a recent monkey experiment that used random-dot motion stimuli with a slightly different paradigm (Nomoto et al., 2010). In the present study, we did not model the activity of individual DA neurons. Instead, we directly modeled the DA levels at the Cx-CD synapses. Specifically, the DA system consisted of the following four processes:

(1) Estimated reward probability (reward prediction) as a function of motion coherence. After training, the subjects were able to estimate the probability of receiving a reward based on the motion stimuli. In order to simplify the computation of the model, instead of estimating the reward probability from the outcome of past trials, we used a preset function to estimate reward probability *p*_est_(*c*′):
  (1) $$p_{\text{est}}(c^{\prime })=1-\frac{1}{2}e^{-{\left(c^{\prime }/\beta \right)}^{\alpha }},$$
  where α = 1 and β = 0.047. The function has been used to fit the simulated performance data that are produced by the same attractor network model (Lo and Wang, 2006). The parameter β was determined by fitting the equation to the overall performance of the model. We tested the model with different values of β and found that the behavioral outcome of the circuit model was not sensitive to the particular choice of the parameter. These results suggested that the model worked without the need to accurately estimate the reward probability.

(2) Phasic DA response to the motion stimulus. DA levels exhibit a phasic increase after the onset of the motion stimulus based on the estimated reward probability as we discussed above. The difference (in arbitrary units) between the peak DA level of the phasic responses and the neutral level, at which no synaptic weight change can be induced, correlates with the estimated reward probability *p*_est_(*c*′) and is given by:
  (2) $$\Delta {\text{DA}}_{\text{est}}=c_{\text{est}}\times 0.15\times r.$$
  where
  (3) $$r=\frac{\left(p_{\text{est}}(c^{\prime })-0.5\right)}{0.5},$$
  and *c*_est_ = 1. *c*_est_ represents the sensitivity of the DA system to reward estimation and can be tuned to test how the sensitivity influences the decision behavior. Because the value of *p*_est_(*c*′) falls between 0.5 and 1, the value of *r* falls between 0 and 1. Combining the equations for ΔDA_est_ and *p*_est_(*c*′) described above, we determined that the peak level of the phasic DA response to the stimulus onset followed a monotonically increasing function of the motion strength *c*′ (Figure 2B, top panel). Based on the observation of a quick increase in DA levels following DA neuron activity and slow reuptake (Suaud-Chagny et al., 1995), we further assumed that, after the onset of the motion stimulus, DA levels increase to their peak level exponentially (time constant = 10 ms) for 100 ms and then drop to the baseline level (−0.2) exponentially (time constant = 150 ms).

(3) Phasic DA response to reward delivery or absence. After the decision is made and the reward is received or not received, the DA neurons exhibit phasic responses that correlate with the reward prediction error. We modeled the phasic changes of the DA levels (in arbitrary units) with two separate functions. When a reward is delivered (100 ms after the choice is made), the DA levels increase briefly to a peak level. The difference between the peak level and the neutral level is given by
  (4) $$\Delta {\text{DA}}_{r}=c_{\text{err}}\times 0.71\times (1-r).$$
  When a reward is not received at the end of a trial, the DA levels decrease briefly to a lower boundary level. The difference between this lower boundary level and the neutral level is given by
  (5) $$\Delta {\text{DA}}_{nr}=c_{\text{err}}\times -1.5\times r-0.6.$$
  *c*_err_ = 1 in both equations. *c*_err_ represents the sensitivity of the DA system to the reward prediction error and is changed when we test how the sensitivity affects the decision behavior. Considering that *r* depends in a linear fashion on the estimated reward probability that positively correlates with the motion strength *c*′, we determined that ΔDA_r_ monotonically decreases with *c*′ while |ΔDA_*nr*_| monotonically increases with *c*′ (Figure 2B, middle and bottom panels). Following our assumption**s** for DA release and reuptake, we here assumed that, after the reward is delivered, DA level**s** increase to their peak level ΔDA_*r*_ exponentially, with a time constant of 10 ms, for a period of 100 ms and then drop to baseline exponentially, with a time constant of 150 ms. In the absence of a reward, the DA level**s** reduce to their lower boundary level ΔDA_*nr*_ exponentially, with a time constant of 150 ms, for a period of 100 ms and then increase to the baseline level with the same time constant (150 ms). Combining Equations (1)–(5), we determined the temporal profiles of the DA level**s** (Figure 2A): at the stimulus onset, the DA level**s** increase intensely for easy trials but weakly for difficult trials. In contrast, when a reward is delivered in a correct trial, the DA level**s** increase weakly for the easy condition but intensely for the difficult condition. In erroneous trials, the DA level**s** exhibit a deep recession for the easy condition but a shallow recession for the difficult condition.

We note that the DA level changes (ΔDA_est_, ΔDA_*r*_, and ΔDA_*nr*_) and the baseline level discussed above are defined with respect to the neutral level that represents the concentration of DA that does not induce synaptic plasticity (see below).

(4) DA modulated spike-timing dependent plasticity (STDP). Based on the extensive experimental evidence on the dopaminergic modulation of corticostriatal synapses (Fino et al., 2005; Calabresi et al., 2007; Pawlak and Kerr, 2008; Shen et al., 2008; Gerfen and Surmeier, 2011), we hypothesized that the stimuli and rewards modify the strength of Cx-CD synapses in the proposed model through DA-modulated STDP. A recent study has shown that, for D1 receptor-expressing striatal medium spiny neurons, high DA levels result in synaptic facilitation in the condition of positive spike timing (pre-synaptic spikes precede post-synaptic spikes), whereas low DA levels produce synaptic depression for both positive and negative spike timing (Shen et al., 2008). Based on these findings, we assumed that the STDP kernel (synaptic weight change as a function of spike timing) in our model is described by the following equations:
  (6) $$\Delta w=w_{\max}\times \exp\left(\frac{-\Delta t}{\tau }\right)\times \Phi ,$$
  (7) $$\Phi =\frac{2}{1+e^{-k\Delta DA}}-1,$$

where Δ*w* is the weight change of Cx-CD synapses, *w*_max_ sets the maximum of Δ*w*, (τ = 30 ms) is the decay constant of STDP influence, and Δ*t* represents the timing difference between the post-synaptic spike and the pre-synaptic spike, and is defined by:

Δ*t* = time of last post-synaptic spike—time of last pre-synaptic spike.

Φ is a term describing the influence of ΔDA over the amplitude of the synaptic weight change with a parameter (*k* = 1). ΔDA is defined as the relative DA concentration with respect to the neutral level. A positive ΔDA results in a positive Φ, which leads to synaptic facilitation (Δ*w* > 0), whereas a negative ΔDA leads to synaptic depression (Δ*w* > 0). At the neutral level (ΔDA = 0), no synaptic weight change occurs. In addition, *w*_max_ takes different values for different spike-timing conditions and DA level conditions. Specifically, *w*_max_ = 5.0 × 10^−4^, 2.0 × 10^−4^, 0, or 2.0 × 10^−4^ (nS) for Δ*t* > 0 and Φ > 0, Δ*t* > 0 and Φ < 0, Δ*t* < 0 and Φ > 0, or Δ*t* < 0 and Φ < 0, respectively. The setting leads to a STDP kernel that produces synaptic facilitation for positive spike timing (Δ*t* > 0) at high DA levels (Φ > 0) but synaptic depression for both positive and negative timing at low DA levels (Φ < 0).

After combining Equations (1)–(7), we obtained a reward- and stimulus-dependent STDP kernel (Figures 2C,D). After the stimulus onset, a strong motion stimulus results in a prediction of high reward probability that also results in strong synaptic facilitation for positive spike timing, while a weak motion stimulus results in a prediction of low reward probability that results in weak synaptic facilitation (Figure 2C). After the decision is made, if the motion stimulus was strong, the subject strongly expects a reward. Therefore, the delivery of the expected reward only results in weak facilitation. In contrast, if the reward is not delivered (due to a wrong choice), the unexpected reward absence leads to a strong depression (Figure 2D). However, if the motion stimulus is weak, the subject does not highly expect a reward, and, therefore, a reward delivery induces a strong facilitation while reward absence produces a weak depression (Figure 2D). Following the responses to the stimulus presentation and reward delivery/absence, the DA levels decay or increase to the baseline level, DA_*b*_, which is set to be −0.2 with respect to the neutral level. The baseline DA levels result in a weak depression that slowly brings down the synaptic strength during the intertrial intervals (ITI).

Finally, we considered the empirical observations in which the relative change in the synaptic strength was smaller for a stronger synaptic strength when the synapse was facilitated, whereas the relative change remained constant when the synapse was depressed (Bi and Poo, 1998; van Rossum et al., 2000). In order to capture this property, for every pre/post-synaptic spike pair, if the resulting synaptic weight change was facilitation (Δ*w* > 0), we used a simple additive rule, Δ*g* = *g*_*t* + 1_ −*g*_*t*_ = Δ*w*, to update the efficacy *g* of the Cx-to-CD synapses, whereas if the resulting weight change was depression (Δ*w* < 0), we used a multiplicative rule, Δ*g* = −*g* |Δ*w*|. The additive updating rule resulted in a percentage change that reduced with the synaptic strength (Δ*g*/*g* = Δ*w*/*g*), while the multiplicative rule resulted in a constant percentage change (Δ*g*/*g* = |Δ*w*|) (van Rossum et al., 2000). In the model, the efficacy *g* of the Cx-to-CD synapses is updated after each pre- or post-synaptic spike based on the STDP kernel Equations (6) and (7) with the multiplicative or additive rules stated above. The updates of the synaptic efficacy are long-term and do not change until the next pre- or post-synaptic spike.

We noted that, due to the noisy background input that each neuron receives, the weights of the Cx-CD synapses develop heterogeneity in the population under the effects of STDP (Kepecs et al., 2002). In order to avoid the complications that arise from the heterogeneity and that were outside of the scope of the present study, we implemented a “*mean-field*” approach in which each Cx-CD synapse followed a common synaptic strength that was updated based on the pre- and post-synaptic spikes that were pooled from every Cx neuron and every CD neuron.

### Reward rate function

We quantified the quality of the decision by calculating the conventional (objective) reward rate and a subjective reward rate, which was weighted by the subject's preference for speed or accuracy. The objective reward rate was defined as the average amount of reward received per unit time (in s) (Gold and Shadlen, 2002):

(8) $$R_{o}=\frac{p_{c}}{T_{\text{ITI}}+T_{R}+(1-p_{c})T_{p}}=\frac{1-p_{\text{err}}}{T_{\text{ITI}}+T_{R}+p_{\text{err}}T_{p}},$$

where *p*_*c*_ represents the performance (percentage correct), T_ITI_ is the inter-trial interval (500 ms), *T*_*R*_ is the mean reaction time, *T*_*p*_ (2500 ms) is the penalty period that is appended to the end of every error trial, and *p*_err_ is the percentage error (= 1 − *p*_*c*_). The objective reward rate *R*_*o*_ forms an inverted U-shaped curve as a function of the decision threshold (Gold and Shadlen, 2002) or the Cx-CD synaptic strength (Lo and Wang, 2006). Ideally, a subject should try to find the optimal threshold or synaptic strength that maximizes *R*_*o*_ (peak of the inverted U-shaped curve) in order to receive as much reward as possible in a given period of time. However, various studies have shown that individual subjects, including humans and animals, may favor speed over accuracy or vice versa (Kay et al., 2006; Rinberg et al., 2006). Therefore, the subjects may not seek to maximize *R*_*o*_ during the decision but rather to speed up or to increase their accuracy. In order to quantify such behavior, we need to measure the subjects' degree of preference for speed or accuracy. A number of studies have quantified the tendency for favoring accuracy over maximizing the reward rate for human subjects (Maddox and Bohil, 1998; Bogacz et al., 2006). We extended this idea by including the tendency for favoring speed and constructed a subjective reward rate function:

(9) $$R_{s}=\frac{1-k_{a}p_{\text{err}}}{T_{\text{ITI}}+k_{s}(T_{R}+p_{\text{err}}T_{p})}$$

where *k*_*a*_ and *k*_*s*_ are the weighting factors (with values between 0 and 1) representing the degrees of preference for accuracy and speed, respectively. *R*_*s*_ also forms an inverted U-shaped function of the Cx-CD synaptic strength with different peak locations for different values of (*k*_*a*_, *k*_*s*_) (Figures 5A,B). Therefore, if the Cx-CD synaptic strength of a subject converges to a specific value that corresponds to the peak location of *R*_*s*_ with a given (*k*_*a*_, *k*_*s*_), we can say that the subject's decisions were characterized by an optimization that was weighted by their specific preference represented by the factors *k*_*a*_ and *k*_*s*_. A large *k*_*a*_ (*k*_*a*_ > *k*_*s*_) indicates that the subject is sensitive to the change in accuracy due to errors, whereas a large *k*_*s*_ (*k*_*s*_ > *k*_*a*_) indicates that the subject is sensitive to the change in the overall trial time due to slow decisions or additional penalty periods. We can recover the conventional (objective) reward rate Equation (8) by setting *k*_*a*_ = *k*_*s*_ = 1. In the present study, we showed that the optimal objective reward rate, *R*_*o*_, for a given speed-accuracy preference (*k*_*s*_, *k*_*a*_) can be achieved in our model by tuning DA-related parameters.

## Results

### Dependence of the synaptic weight on the trial difficulty and trial outcome

**Figure 3:**
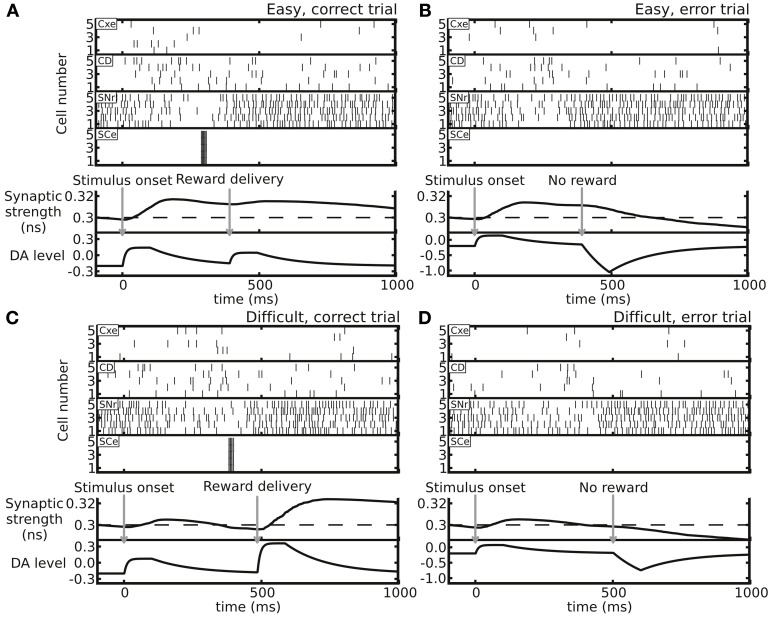
**Example trials showing distinct neuronal activity and resulting Cx-CD synaptic weights in the following four conditions. (A)** An easy trial with a correct decision. **(B)** An easy trial with an incorrect decision. **(C)** A difficult trial with a correct decision. **(D)** A difficult trial with an incorrect decision. The top four spike rastergrams in each panel display the spike activity of neurons in the Cxe, CD, SNr, and SCe in the side that corresponds to the correct choice. The bottom two plots in each panel indicate the time courses of Cx-CD synaptic strength and DA levels (in arbitrary units). In general, correct trials result in synaptic facilitation, while error trials depress synapses.

We tested the changes in synaptic weight in different trial conditions (Figure 3). In an easy trial (strong motion strength) in which the subject made a correct choice, the DA levels responded strongly to the stimulus onset due to the large estimated reward probability. However, the DA levels responded to the reward delivery only weakly because the reward was highly anticipated (Figure 3A). In contrast, in a difficult trial with a correct choice, the DA levels responded to the stimulus onset weakly, while the levels responded to the reward delivery strongly due to the less anticipated reward (Figure 3C). The overall effect of the synaptic weight changes for a correct choice in both easy and difficult conditions was facilitation. When the subject made an incorrect choice, even though the DA increase in response to the stimulus onset slightly facilitated the synapse, the absence of reward reduced the DA levels, which resulted in an overall synaptic depression (Figures 3B,D).

**Figure 4:**
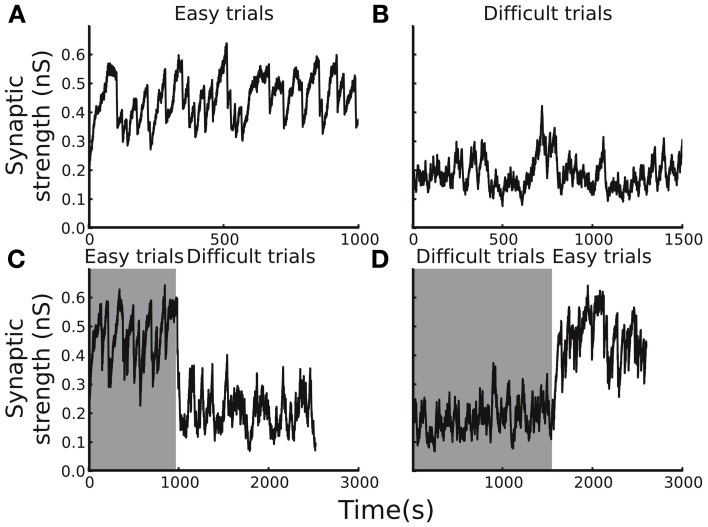
**Average Cx-CD synaptic strengths remain stable but exhibit different levels for different task conditions. (A)** The time course of the synaptic strength in a block of trials with the easy condition. The synaptic strength converged to a range between 0.3 and 0.6 nS. **(B)** The time course of the synaptic strength in the difficult condition. The synaptic strength converged to a range (between 0.1 and 0.4 nS), which is lower than that in the easy condition. **(C)** When the trial condition switched from easy to difficult, or **(D)** from difficult to easy, the synaptic strength quickly converged to new range that is consistent with those shown in the **(A,B)**. The results show that the model exhibits stable and consistent adaptations to changes in task difficulty.

The plasticity rule that was used in our model was spike-time based, and, therefore, the synaptic weight change was sensitive to the variability of the spiking timing, which could be large in noisy neuronal environments. As a consequence, whether the synaptic strength was able to reach a stable level in a block of trials and whether the level of the stable synaptic strength depended on the task difficulty remained in question. To address the question, we tested the circuit model with blocks of trials with easy or difficult conditions. We set the initial Cx-CD synaptic strength to be 0.1 nS and found that, for the easy condition, the strength quickly reached a range of 0.3–0.6 nS, and the range remained stable afterwards (Figure 4A). In contrast, testing the model with the difficult condition also resulted in a stable but different range of Cx-CD synaptic strength (~0.1–0.3 nS) (Figure 4B). The results showed that the model was able to operate in stable but different ranges of Cx-CD strengths for different task conditions. Because the decision threshold is monotonically decreased with increased Cx-CD strength (Lo and Wang, 2006), the stronger Cx-CD strength in the easy compared to the difficult conditions indicated that the circuit model reduced the decision threshold for faster responses when the task was easy while it raised the decision threshold for a better performance when the task was difficult.

### Adaptation to the change in task difficulty

We showed that the circuit model tuned itself and operated at different levels of Cx-CD strengths when tested with trials involving either easy or difficult conditions. However, in natural environments, the task conditions may vary with time. Therefore, we asked whether the circuit model was able to adapt to environmental changes by testing the model with a sudden switch of the task condition from easy to difficult and vice versa (Figures 4C,D). We found that the circuit model was able to quickly change its Cx-CD strength after the task condition switched. Specifically, the Cx-CD strength decreased when the task condition changed from easy to difficult, while it increased when the change reversed. These results indicated that the circuit model was able to respond to the increased task difficulty by slowing down the decision speed, while it responded to decreased task difficulty by speeding up. Furthermore, we calculated how long the model took to complete the transition after the switch of the task condition. To this end, we counted the number of trials it took for the Cx-CD synaptic strength to reach the new average strength after the switch. We found that it took 30 ± 25 trials for the easy to difficult switch and 60 ± 28 trials for the difficult to easy switch.

### Decision optimization

**Figure 5:**
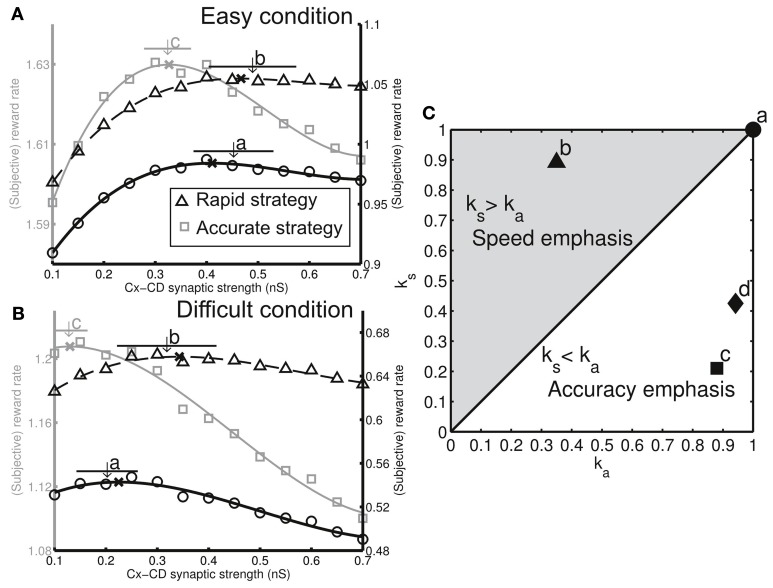
**Different decision strategies (characterized by favoring speed or accuracy) can be realized by tuning three dopamine-related parameters**. **(A)** Each decision strategy can be represented by a subjective reward rate curve in the easy condition. We plotted curves for three different decision strategies (gray curve: left ordinate, solid and dashed black curves: right ordinate). **(B)** The same three decision strategies show shifted subjective reward rate curves in the difficult condition. We performed simulations for the model with three different model settings and found that each of the settings forms specific ranges of Cx-CD synaptic strengths in both task conditions (**a**, original as in Figures 3, 4, **b**, enhanced responses to reward presentation/absence, and **c**, reduced baseline DA level. The arrows indicate the mean synaptic strength and horizontal bars represent the standard deviation). We found that each of the model settings corresponded to the optimization for a specific decision strategy (*k*_*a*_, *k*_*s*_). The x's indicate the peak locations of the curves. **(C)** Corresponding decision strategies for the three model settings [**a–c** in **(A,B)**] shown on a (*k*_*a*_, *k*_*s*_) plane. In addition, we tested the model by reducing the response of DA level to the stimulus presentation and found that the setting resulted in an accuracy-emphasis strategy (labeled by **d**), which is similar to that of the setting **c**.

The adaptive decision behavior shown in Figures 4C,D led to the fundamental question whether the adaptation was optimal. More specifically, does the decision strategy (the specific Cx-CD strength chosen by the circuit model) maximize the reward rate in both easy and difficult conditions? Our earlier study showed that the (objective) reward rate, as a function of Cx-CD strength, forms an inverted U-shaped curve. Therefore, there is an optimal Cx-CD strength that corresponds to the peak of the curve that gives rise to the maximum reward rate (Lo and Wang, 2006). We further found that the inverted U-shaped curve shifts or the optimal Cx-CD strength changes with different task conditions (Lo and Wang, 2006). Therefore, in the present study, we asked whether the model was able to automatically converge on the optimal Cx-CD strength when the task condition changed from easy to difficult. Indeed, we found that, when the task condition was easy, the model converged on the optimal Cx-CD strength (Figure 5A, thick black curve, *k*_*s*_ = *k*_*a*_ = 1). After we switched the task condition to difficult, the model quickly changed its Cx-CD strength to a lower range that was optimal in the difficult condition (Figure 5B, thick black curve, *k*_*s*_ = *k*_*a*_ = 1).

One may argue that the optimization depends on the choice of the model parameters and ask what would happen if we chose different parameters. Below, we show that with different values of dopamine-related parameters our model still reached optimal decisions that were characterized by different weights for speed and accuracy.

### Subjective reward rate

As discussed in the Introduction and Materials and Methods, human and animal subjects may not be ideal decision-makers that maximize their objective reward rate Equation (8). Rather, some individuals may favor more accurate decisions, while others may favor faster decisions. In other words, subjects may have their own *subjective* senses of optimization that are weighted by their preferences for speed or accuracy. We proposed a subjective reward-rate Equation (9) that can be used to quantify such preferences that are characterized by the values of the speed-weighting factor, *k*_*s*_, and the accuracy-weighting factor, *k*_*a*_.

We addressed the two following fundamental questions. First, what are the neuronal substrates that influence an individual's preference for speed or accuracy? This question is equivalent to finding the neuronal parameters that achieve maximum subjective reward rates for a given combination of *k*_*s*_ and *k*_*a*_. Second, does the adaptive decision behavior remain optimal in terms of subjective reward rate when the task condition changes? This question is equivalent to asking, if a subject maximizes the subjective reward rate for given *k*_*s*_ and *k*_*a*_ in a task condition, does the subject still achieve the maximum subjective reward rate (for the same *k*_*s*_ and *k*_*a*_) when the task condition changes? We addressed the questions with the following procedure:

1. For each grid point on the (*k*_*s*_, *k*_*a*_) space (ranged from 0 to 1 with a grid space of 0.05), we calculated the subjective reward rate *R*_*s*_ Equation (9) for both easy and difficult conditions and then found the optimal Cx-CD synaptic strength that maximized *R*_*s*_ in each condition.
2. We tuned the neuronal parameters, performed simulations, and then calculated the mean Cx-CD synaptic strengths for each task condition (easy or difficult). Here, we selected three DA-related parameters for tuning: (1) *c*_est_ in Equation (2), representing the magnitude of the DA responses to the stimulus onset (estimate of reward probability); (2) *c*_err_ in Equations (4) and (5), representing the magnitude of the DA responses to the reward delivery or absence (reward prediction error); and (3) The baseline DA level, DA_*b*_. For each set of parameters (*c*_est_, *c*_err_, and DA_*b*_) we found the mean and the standard deviation of the Cx-CD strengths for each task condition.
3. Finally, by combining the results from 1 and 2, we looked for the (*k*_*s*_, *k*_*a*_) that gave rise to the optimal Cx-CD strengths that matched the simulated mean Cx-CD strengths for each parameter set (*c*_est_, *c*_err_, and DA_*b*_) for both the easy and difficult conditions. We noted that the matched *k*_*s*_ and *k*_*a*_ may not fall exactly on a grid point in the (*k*_*s*_, *k*_*a*_) space. Therefore, we first located an area in the (*k*_*s*_, *k*_*a*_) space with the enclosed grid points that had optimal Cx-CD strengths very close to the simulated mean strengths (typically within 0.5 standard deviation). Then we had the center point of this area represent the matched (*k*_*s*_, *k*_*a*_).

We were able to identify the corresponding *k*_*s*_ and *k*_*a*_ for each model setting set that we tested (Figures 5A,B). We plotted the results for four representative settings. Setting **a** corresponded to the original setting (used in Figures 3, 4) in which *c*_est_ = *c*_err_ = 1 and DA_*b*_ = −0.2. Setting **b** represented enhanced responses to reward delivery and absence (*c*_err_ = 1.8). In setting **c**, we reduced the baseline DA level (DA_*b*_ = −0.25). Setting **d** represented the weakened responses to stimulus presentation (*c*_est_ = 0.9). The results showed that the different model settings represented the optimizations for different decision strategies (*k*_*s*_ and *k*_*a*_ combinations). Moreover, the circuit remained optimal for the same strategy when the task conditions changed.

**Figure 6:**
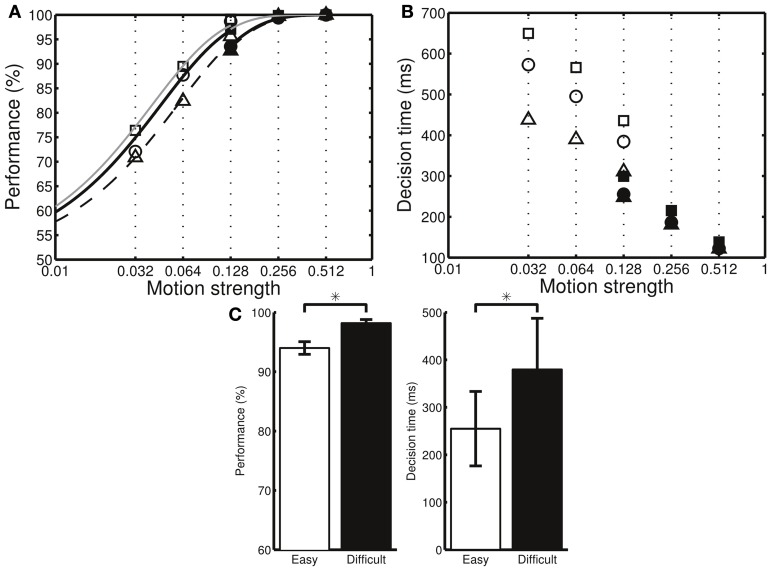
**The three different model settings (a–c in Figure 5) result in different behavioral performances**. **(A)** The percentage correct is higher in the setting that emphasizes accuracy (squares: setting c, circles: setting a, triangles: setting b). Filled and open symbols indicate the data for the difficult and easy conditions, respectively. To help visualize the differences, the data were fitted separately in the two conditions using $p(c^{\prime })=1-\frac{1}{2}e^{-((c^{\prime })/\beta )}$ where β is a fitting parameter. **(B)** The mean decision times for the same model settings. The mean decision times are smaller in the setting that emphasizes speed. Both easy and difficult conditions include the stimulus motion strength of *c*′ = 12.8%. However, due to the decision adaptation, the neural circuit converges to different levels of Cx-CD synaptic strengths in the two task conditions. As a result, the behavioral performances for the same motion strength of *c*′ = 12.8% in the two task conditions are different. **(C)** By analyzing the data from setting a, we found that the percentage correct (left panel) is higher, while the mean decision time (right panel) is larger in the difficult than in the easy conditions. ^*^p < 0.05.

Next, we examined how the neuronal parameters, *c*_est_, *c*_err_, and DA_*b*_, affected a subject's preference toward either speed or accuracy. We found that the simulated subject favored accuracy (*k*_*a*_ > *k*_*s*_) when we reduced the sensitivity, *c*_est_, of the DA system to the estimate of reward probability as well as reduced the baseline DA level, DA_*b*_. In contrast, the simulated subject favored speed (*k*_*s*_ > *k*_*a*_) if we increased the sensitivity, *c*_err_, to the reward prediction error (Figure 5C). We further analyzed the simulated result at the behavioral level and verified that, with a preference to accuracy (*k*_*a*_ > *k*_*s*_), the percentage correct was larger but the decisions were slower than those in the ideal decision cases (*k*_*a*_ = *k*_*s*_ = 1). In contrast, with a preference to speed (*k*_*s*_ > *k*_*a*_), the decisions were faster but the error rate was higher than those in the ideal decisions (Figures 6A,B).

### Differential responses to the same stimulus due to adaptation

In our task, the subjects were presented with stimuli with motion coherence levels of *c*′ = 3.2, 6.4, or 12.8% in the difficult condition and *c*′ = 12.8, 25.6, or 51.2% in the easy condition. The task design was unique in that the subjects encountered stimuli with *c*′ = 12.8% in both conditions. Due to the adaptive behavior that brings the Cx-CD synaptic strength to different levels between the two conditions, we found that, for the same motion strength (*c*′ = 12.8%), the model circuit performed better in the difficult than in the easy conditions (Figure 6C). This observation provided a behavioral assessment that allowed us to easily assess whether a subject exhibited adaptive behavior when the task difficulty changed. We noted that this was a general result of adaptive behavior and was not specific to our model.

### Roles of the phasic responses of DA levels in decision adaptation

**Figure 7:**
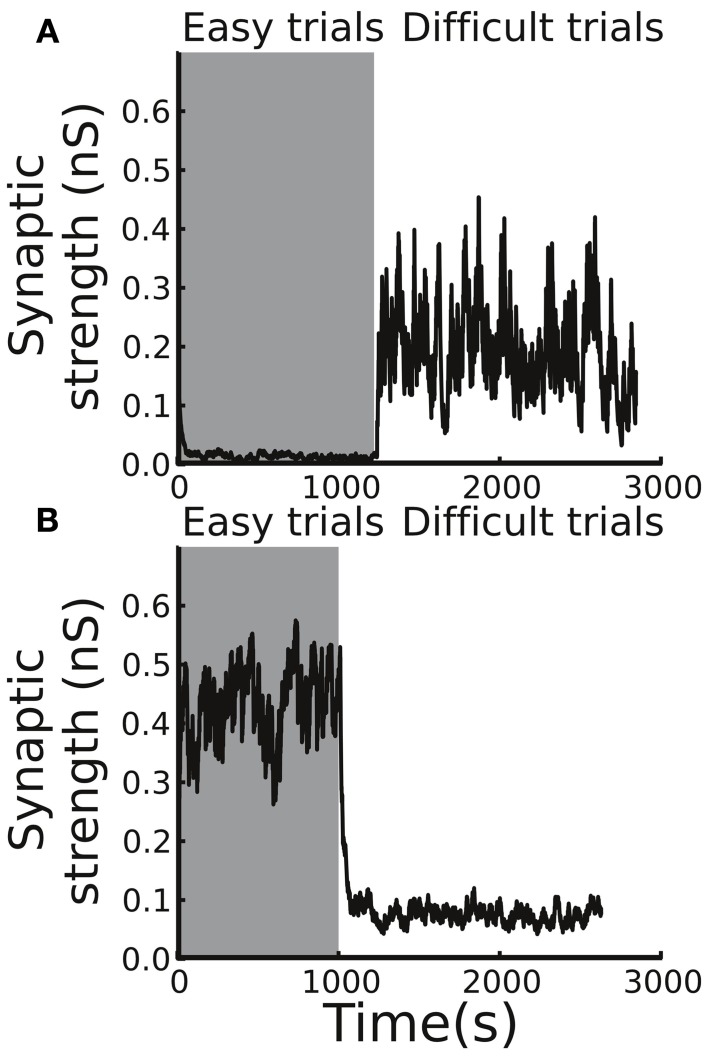
**Different contributions of the phasic responses to decision optimization**. **(A)** By removing the response of the DA system to the stimulus presentation, the circuit model was not able to maintain a stable Cx-CD synaptic strength in the easy condition. **(B)** In contrast, when we removed the response of the DA system to the reward delivery/absence, the model failed when the task condition switched to difficult.

It is important to test individual contributions of the two phasic DA responses (to the stimulus presentation and to the reward delivery/absence) in the adaptive behavior of decision making. To this end, we performed two sets of simulations. In the first set, we tested the model by removing the DA responses to the stimulus presentation (estimate of reward probability), and, in the second set, we removed the DA responses to the reward delivery/absence (reward prediction error). We found that, without the phasic DA response to the stimulus presentation in the beginning of a trial, even though the Cx-CD strength was able to remain at stable levels in the difficult condition, the strength could not be maintained and quickly dropped to nearly zero in the easy condition (Figure 7A). This was because, in the difficult condition, the stability of the Cx-CD strength mainly relied on the balance between the facilitation that was due to the reward delivery (large positive reward prediction errors) and the depression that was due to the absence of the reward in the error trials and the baseline DA levels. Removing the DA responses to the stimulus presentation did not produce much impact on the circuit. However, in the easy condition, the reward delivery only weakly facilitated the Cx-CD synapses (due to small reward prediction errors), and the facilitation that was induced by the strong DA responses to the stimulus presentation (due to the large estimated reward probability) played a crucial role in maintaining the stability of the Cx-CD synapses. Removing the DA responses to the stimulus presentation destroyed the balance between the facilitation and depression. As a consequence, the circuit was dominated by the depression that occurred when the DA level was at the baseline level and when the reward was not delivered in erroneous trials. In the second set of simulations, we removed the response of the DA levels to the reward delivery/absence and found that Cx-CD strength dropped to nearly zero in the difficult condition but was maintained at a reasonable level in the easy condition (Figure 7B). The imbalance between the facilitation and depression that occurred in the difficult condition was mainly due to the loss of the strong facilitation that was induced by reward delivery. The effect of the loss of depression that was induced by reward absence was relatively minor. These results suggested that both phasic DA responses were crucial for producing proper synaptic strength but that they contributed differently in the easy and difficult conditions. Thus, with only one phasic response, a subject may still be able to achieve optimal decisions in one condition but will fail when the task condition changes.

### Adaptation across different inter-trial intervals and penalty times

**Figure 8:**
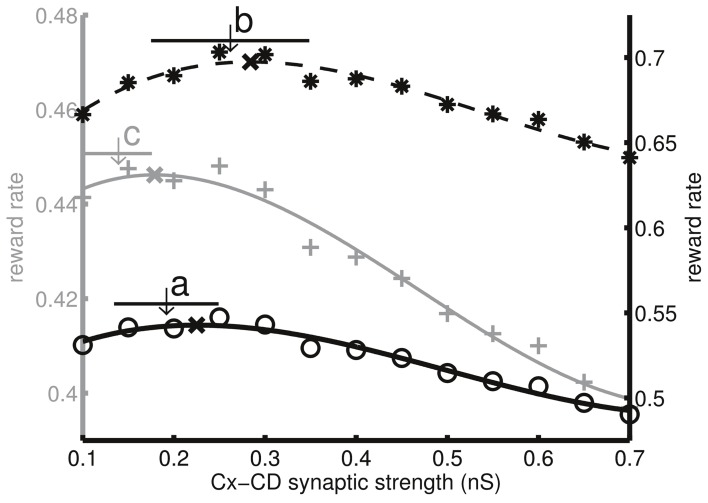
**Decision optimization under different speed settings by changing the intertrial interval (ITI) and the penalty time**. **a**, The original setting (ITI = 500 ms, penalty time = 1,500 ms), **b**, the fast setting (ITI = 200 ms, penalty time = 1200 ms), and **c**, the slow setting (ITI = 800 ms, penalty time = 1800 ms). Circles, pluses, and asterisks indicate the corresponding subjective reward rates (*k*_*s*_ = *k*_*a*_ = 1) as functions of Cx-CD synaptic strength. As the ITI and the penalty times increase, the optimal Cx-CD synaptic strength that maximizes the reward rate reduces. Using the same parameters (set **a** in Figure 5) that optimize the objective reward rate (*k*_*s*_ = *k*_*a*_ = 1) under different difficulty settings, we found that the model also followed the same trend by lowering the average Cx-CD synaptic strength, as indicated by the shifted arrows. The horizontal bars represent the standard deviations of the synaptic strengths in the corresponding conditions.

So far, we only tested the model by changing the task difficulty. We asked whether the same model also optimized decisions under other different task conditions involving, in particular, the temporal aspects of the task setting. The reason was that the subjects' perception to accuracy and time are two of the major factors that influence decision adaptation and optimization. Because we already tested the model by changing the task difficulty, the next objective was to test changing the speed or the pace at which the subjects performed the task. The easiest temporal parameters to manipulate are the ITIs and penalty times. Here, we used the difficult setting (*c*′ = 3.2, 6.4, and 12.8%) and changed the task conditions by adding 300 ms to both ITIs and penalty times as well as by subtracting 300 ms from both of them. The model parameters followed those that were used in Figures 3, 4 (or set **a** in Figure 5). We found that the subjective reward rate curve (*k*_*a*_ = *k*_*s*_ = 1) shifted toward the left as the ITI and the penalty time increased. Similarly, the model also exhibited the same trend by reducing the mean Cx-CD synaptic strength (Figure 8). We noted that a deviation between the simulated synaptic strength and the optimal strength was observed after adding 300 ms (ITI = 800 ms, penalty time = 1800 ms), which characterizes the limitation of the model. A possible solution to the deviation is discussed in the Discussion section.

## Discussion

**Figure 9:**
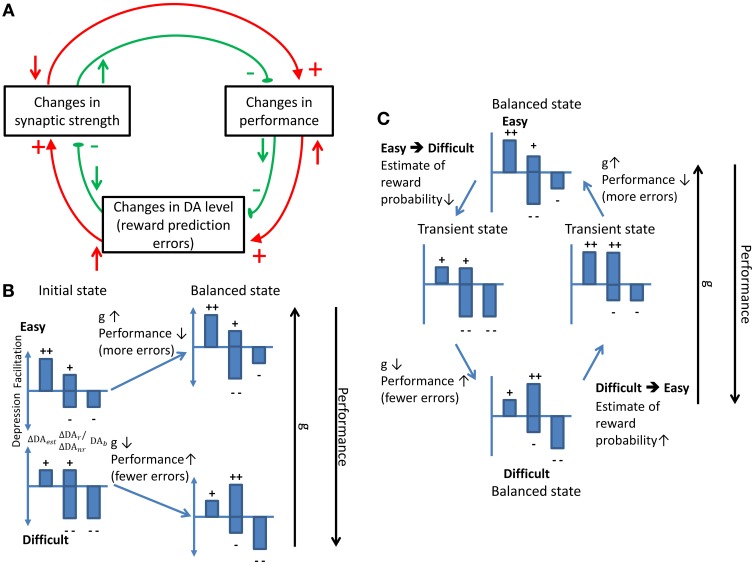
**Schematics showing how stability and adaptation are realized by the dynamic balance between different model components**. **(A)** The stability of synaptic strength under a balanced condition. Any change in the synaptic strength (upper left rectangle) will alter the performance which will in turn change the DA level that leads to an opposite effect on the synaptic strength. The red and green circles indicate the flow of the processes induced by a decrease and an increase in the synaptic strength, respectively. The upward arrows represent an increase in the level of the source components while the downward arrows represent a decrease. Plus and minus signs indicate the facilitating and depressing effects on the target components, respectively. **(B)** The dynamic balance leads to a stronger synapse in the easy than in the difficult conditions. In each small bar chart, the left bar indicates the response of DA that represents the estimate of the reward probability (ΔDA_est_). The two middle bars indicate the response of DA to the reward delivery (ΔDA_*r*_) (the upper one) and to the reward absence (ΔDA_*nr*_) (the lower one). The bar on the right represents the baseline DA level (ΔDA_*b*_). The plus signs indicate the facilitating effect which is against the depressing effect indicated by the minus signs. The summation of the plus and the minus signs indicates the direction of change in the synaptic strength. When the task condition is easy, the high ΔDA_est_ level increases the synaptic strength which reduces the performance. As a consequence, ΔDA_*nr*_ becomes stronger and the synaptic facilitation stops. In contrast, the difficult task condition initially depresses the synapse. The depression leads to improved performance that induces more facilitating effect and, thus, the initial synaptic depression stops. **(C)** The balance shifts and re-established when there is a change in the task conditions. Such a dynamic balance underlies adaptive decision making.

In summary, we proposed a neural circuit model that was endowed with dopamine-modulated plasticity and that remained optimized (maximum subjective reward rates) in adaptation to changes in the task conditions. The ability is realized by the effects of the following three DA components: (1) responses of DA to the stimulus presentation (estimate of reward probability), (2) responses of DA to the reward delivery/absence (reward prediction error), and (3) the baseline DA levels that causes slow synaptic depression. Balance between the facilitating and depression effects caused by these components is crucial for establishing a stable decision strategy (Figure 9A).

When a balance between the DA components is reached, the DA components that produce synaptic depression are compensated for by ones that facilitate synapses. Hence, changing the task condition results in changing the performance and tilting the balance between facilitation and depression. As a consequence, the adaptive behavior occurs when the Cx-CD synaptic strength shifts until a new balance is reached. We illustrate the process with an example. Assuming that the Cx-CD synaptic strength is in a stable state in the easy condition and if the task condition suddenly switches to difficult, the DA responses to the stimulus onset are weakened due to the lower estimated reward probability. The changes lead to less synaptic facilitation. In addition, the number of erroneous trials increases and the average trial time is prolonged (due to the punishment period). Both changes induce synaptic depression and effectively raise the decision threshold. As the threshold rises, the performance improves and the percentage of correct trials increases. The performance improvement leads to more positive reward prediction errors (more rewards than expectation) which facilitate the synapses. As a consequence, the depression and facilitation effects reach the balance and the Cx-CD strength is stabilized at a new and weaker level in the difficult condition. A similar argument can be made for the case in which the task condition switches from difficult to easy (Figures 9B,C).

By defining the speed and accuracy factors (*k*_*s*_ and *k*_*a*_) for the decision behavior, we were able to identify DA-related parameters that accounted for the preference for speed or accuracy. Below, we discuss how the key DA components work to produce different optimal strategies during the decision process.

### Baseline DA level

In the model, the baseline DA level is slightly below the neutral level and results in a gradual decaying Cx-CD synaptic strength during the course of a trial and between the ITIs. Therefore, reducing the baseline DA level produces a faster decay in the Cx-CD synaptic strength, which leads to an increased decision threshold that improves the accuracy. A better accuracy produces more synaptic facilitation that balances with the strong synaptic depression caused by the lower baseline DA level. As a result, a lower DA level results in a better performance.

### DA responses to stimulus presentation

If we reduce the magnitude of the response, the synapses become more depressed, and, hence, the decision threshold increases. The change results in a higher percentage of correct decisions and reduces the synaptic depression that is caused by erroneous responses. As a consequence, the balance between facilitation and depression is reestablished but with a better decision accuracy (an accuracy-emphasis strategy).

### DA responses to reward delivery/absence

The responses of DA to reward delivery/absence induce synaptic facilitation in correct trials and depression in erroneous trials. However, the overall facilitating effect is stronger than the depressive one. Therefore, if we increase the magnitude of the DA responses, the net effect is to facilitate the synaptic strength. The change produces a lower decision threshold which leads to a faster decision or a higher error percentage. As a consequence, the balance between facilitation and depression is reestablished but with a faster decision (a speed-emphasis strategy).

The adaptive behavior results in a general prediction that can be tested experimentally. At the behavioral level, the model predicts that the performances (percentage correct) at the motion strength of *c*′ = 12.8% are different between the easy block and the difficult block. The difference provides a quick and easy way to assess the existence of the adaptive behavior in decisions. Furthermore, the model suggests that the decision strategy (speed or accuracy emphasis) can be characterized by the accuracy factor *k*_*a*_ and the speed factor *k*_*s*_. Although *k*_*a*_ and *k*_*s*_ are not directly measurable, it is possible to determine a subject's *k*_*a*_ and *k*_*s*_ indirectly by comparing the measured performance and reaction times with the proposed model.

In addition to the behavioral predictions discussed above, our model also makes specific predictions at the neuronal level. Specifically, the model suggests possible neural mechanisms that account for the intersubject and interspecies differences in decision strategies. For example, some animals make quick decisions rather than slower but more accurate decisions (Chittka et al., 2009). Apart from the issues in experimental design (Rinberg et al., 2006; Chittka et al., 2009) and in the differences between sensory modalities, one possible explanation is that the DA systems in animals respond strongly to the presentation of reward delivery/absence and, hence, shift the decision behavior to a speed-emphasis state. If a species, such as humans, tends to emphasize accuracy and make slow decisions, they may have relatively lower DA baselines that enhance the gradual Cx-CD depression and produce an overall weaker Cx-CD synaptic strength or a higher decision threshold. Alternatively, according to our model, the tendency can be accounted for if the subjects have weaker responses to the stimulus presentations.

Due to the lack of experimental data, we did not distinguish the relative contributions of the pre- and post-synaptic factors to the changes in the synaptic strength. According to Equations (6) and (7), the levels of the synaptic strength are affected by Δ*w*, which is proportional to the multiplication between *w*_max_ and ϕ, which is a function of the dopamine levels (ΔDA). Physiologically, *w*_max_ characterizes the post-synaptic factors while ϕ corresponds to the pre-synaptic effect at the DA neuron side. Mathematically, the particular choice of the *w*_max_ values is not important as it can be compensated by changing ϕ. In the present study, we arbitrary set the values for *w*_max_ and then we tuned the magnitudes of DA responses, which effectively changed ϕ, to produce desired decision behavior. Detailed neurophysiological experiments are required to identify the relative contributions of the pre- and post-synaptic factors so that we can adopt more realistic values for *w*_max_ and ϕ.

For the sake of simplicity, we did not include in the proposed model the neural circuit that estimates the reward probability for each stimulus motion strength based on the past trial outcomes. Instead, we used a preset function Equation (1) of reward probability vs. stimulus motion strength. It will be interesting to construct such an estimator circuit in future studies as this circuit is useful in studying the learning process of decision adaptation. A possible solution is to implement a spiking and biologically realistic version of the value estimator as described in Rao (2010). Furthermore, the simulated average Cx-CD synaptic strength was slightly stronger in the easy condition and weaker in the difficult condition or the high ITI + penalty time conditions than the optimal values that were suggested by the objective reward rate curve (Figures 5A,B, 8). We expect that the small deviations could be corrected for if we implement an estimator circuit that can more accurately estimate the performance based on the trial history.

Several computational models for flexible decision behavior may relate to our work. In a firing-rate network model that exhibits rapid threshold tuning, researchers have demonstrated that their model is able to quickly converge to the optimal threshold that maximizes the objective reward rate under different task environments (Simen et al., 2006). However, the network needs prior knowledge about the reward rate-threshold relationships across different task environments, such as varying ITIs or varying difficulties. In contrast, our model does not need such information. Instead, the model requires a rough estimate of performance as a function of difficulty Equation (1), which is independent of the task environment. Compared to the reward rate-threshold relationships, performance as a function of difficulty seems to be the information that is more naturally and easily learned by subjects during training. This argument needs to be tested experimentally. Some other rate-based models have focused on action selection and executive control in BG circuits involving direct and indirect pathways (Cohen and Frank, 2009; Wiecki and Frank, 2013). The inclusion of the indirect pathway is a reasonable choice in their models as it is the pathway that has been associated with inhibitory control, the enhancement of action precision, or the avoidance of aversive stimuli (Hikosaka et al., 2000; Jiang et al., 2003; Hikida et al., 2010). Whether the indirect pathway plays a crucial role in the adaptation and optimization of perceptual discrimination tasks requires further tests. Furthermore, our model focuses on spiking neurons with detailed dopamine dynamics and STDP and is therefore able to provide experimental testable predictions on the correlation between cellular level factors and behavior performance.

We noted that a recent primate study has demonstrated that frontal-eye-field neurons change their ramping rates in a visual search task when the monkeys are cued for different decision speeds (Heitz and Schall, 2012). Their result suggested the possibility of another neuronal mechanism (other than changing the decision threshold) that might underlie the behavior of a SAT. Interestingly, we have recently shown that similar neuronal responses can be observed in the attractor decision model by applying a top–down control with balanced excitation and inhibition (Wang et al., 2013). We have suggested that the two mechanisms of SAT do not exclude each other. Rather, different SAT mechanisms may be implemented by the same subject under different task conditions. It is interesting to integrate the two mechanisms in a single model and to investigate whether the model is able to reproduce a wider range of empirical observations. Furthermore, a recent primate study has shown that neurons in the caudate nucleus encode complex information, including evidence accumulation, evaluation, and choice biases (Ding and Gold, 2010), during a random-dot task. With the combination of a top-down control mechanism (Wang et al., 2013), it is worth exploring how our model is able to reproduce these observations.

In conclusion, we proposed a neural circuit model for decision optimization and adaptation. The model is novel in several of the following aspects. First, the model optimizes (subjectively) the decision not just in a single environment but also in environments with changing task difficulties. Second, the model suggests neuronal substrates that correlate with the decision optimization in different task conditions. Third, the model provides an explanation at the neuronal level for why some subjects favor quick decisions while others favor accurate decisions.

### Conflict of interest statement

The authors declare that the research was conducted in the absence of any commercial or financial relationships that could be construed as a potential conflict of interest.
